# The primary care workforce: a critical element in mending the fractured US health care system

**DOI:** 10.1186/1750-4732-3-11

**Published:** 2009-10-16

**Authors:** Roberto Cardarelli

**Affiliations:** 1 University of North Texas Health Science Center at Fort Worth, Primary Care Research Institute, 855 Montgomery Street, Fort Worth, TX, USA

## Abstract

A focus on the primary care workforce is critical when discussing plans to address the fractured United States health care system. However, we must first address the primary care physician shortage crisis when planning for health care reform which focuses on increasing access to the US population. Initial strategies may include improving reimbursement rates for primary care services, incentivizing medical schools in making primary care training a priority, and developing robust loan-forgiveness programs for those who enter and work in primary care specialties. Planning with congressional representatives about these elements will better ensure sustainable health reform efforts are implemented.

## Introduction

Health care reform has become the topic of discussion in break rooms, across dining room tables, and in barbershops. The issues of increasing health care access, its related costs, and choice of insurance are common threads of debate throughout our communities and among lawmakers. However, we must reflect and ask, "Why must things change in our health care system?" The word "change" naturally evokes panic and healthy discussions, but knowing some fundamental issues we currently face in the US health care system will make us realize that change is needed, and probably inevitable.

The US not only remains a world economic leader, it also claims status as the highest spender in health care [1]. Sixteen percent of the gross domestic product is spent on health care [1]. In 2007 the United States spent approximately $2.3 trillion on health care, of which, 70% and 78% of the cost was attributable to only 10% of the total US population and to those with chronic illnesses, respectively [2,3]. Nonetheless, about 47 million Americans remained uninsured and only 55% received needed care for the leading causes of death and disability [4,5]. Mismanagement of care and undesirable quality of care are core causes to the above statistics, requiring focused efforts to deliver the right care, at the right time, at the right cost.

## Discussion

To improve our fractured health care system, a multifaceted approach will be required. One area that is critical and must be at the forefront is the primary care workforce. The United States is currently experiencing a primary care physician shortage with less than 2% of graduating medical students contemplating a career in internal medicine will choose to remain in general internal medicine [6]. According to a study published by the Robert Graham Center, one of the top reasons medical students do not choose a primary care specialty is its low average annual income [7]. This is understandable when one in four medical students have an average medical school debt above $200,000 [8]. The US health care system over-compensates for high-cost procedures and underpays primary care services, devaluing chronic disease management, preventive care, and the importance of having a personal healthcare provider [9]. However, why must we invest in primary care as it relates to health care quality and cost? Primary care access is proven to reduce hospital admissions and emergency room utilization [10]. Studies have repeatedly shown that primary care physician density is related to reduced overall mortality and health care expenditures [11].

Since current health care reform proposals center upon the notion of increasing access for all Americans, strategies to reverse our primary care physician shortages must immediately take precedence. The solution will be comprehensive and complicated. However, a focus on several key strategies may be fruitful in increasing the number of primary care physicians for the US health care system.

• Increase reimbursement rates, especially from government payors, for services provided by primary care physicians. This will place value on preventive care, management of chronic illnesses, and the coordination of health services. These increases should not be at the expense of Americans, but rather a focus on a redistribution of reimbursements rates between services and specialties to create a more equitable paying system.

• Incentivize medical schools in making primary care a priority within their institutions. For example, state supported medical schools that graduate more than 30% into family medicine and pediatrics should receive additional funding consideration from higher education boards in addition to their formula-driven budgets. Internal medicine is not recommended in the calculation since a large majority of those entering internal medicine residencies continue into sub-specialization tracks [12].

• Develop robust federal loan-forgiveness programs for medical students entering and practicing primary care. For example, recently the 81st Texas Legislature passed House Bill 2154 which will pay up to $160,000 to cover educational loans for primary care physicians who agree to practice for four years in health professional shortage areas. This will directly address the high debts students incur during medical school.

## Conclusion

While these three strategies are stout measures for the health care reform process, they have the ability to dramatically increase the density of the US primary care workforce. For every 1% increase in primary care physicians, we can reduce the number of emergency room visits by over 2,900 in an average-sized metropolitan area [10]. It is critical that we engage our congressional representatives so that impactful and sustainable outcomes will be eventually realized in our country's health care reform efforts.

## Competing interests

The author declares that he has no competing interests.

## Author's Information

Roberto Cardarelli, DO, MPH is an Associate Professor in the Department of Family Medicine and the Executive Director of the Primary Care Research Institute at the University of North Texas Health Science Center. Dr. Cardarelli's research interests include improving primary care practice, physicians disciplined by state medical boards, chronic disease management, evidence-based reviews, and health disparities in point-of-care practice. His main interest in medical care is chronic disease management.
